# Synthesis and Free Radical Scavenging Activity of New Hydroxybenzylidene Hydrazines

**DOI:** 10.3390/molecules22060894

**Published:** 2017-05-29

**Authors:** Frantisek Sersen, Fridrich Gregan, Peter Kotora, Jarmila Kmetova, Juraj Filo, Dusan Loos, Juraj Gregan

**Affiliations:** 1 Institute of Chemistry, Faculty of Natural Sciences, Comenius University in Bratislava, Ilkovicova 6, 842 15 Bratislava, Slovakia; filo@fns.uniba.sk; 2 Department of Chemistry, Faculty of Natural Sciences, Matej Bell University, Tajovskeho 40, 974 01 Banska Bystrica, Slovakia; frido.gregan@gmail.com (F.G.); Jarmila.Kmetova@umb.sk (J.K.); 3 Institute of Process Engineering, Faculty of Mechanical Engineering, Slovak University of Technology in Bratislava, Namestie slobody 17, 812 31 Bratislava, Slovakia; kotora@fns.uniba.sk; 4 Department of Organic Chemistry, Faculty of Natural Sciences, Comenius University in Bratislava, Ilkovicova 6, 842 15 Bratislava, Slovakia; loosdus@gmail.com; 5 Department of Genetics, Faculty of Natural Sciences, Comenius University in Bratislava, Ilkovicova 6, 842 15 Bratislava, Slovakia; 6 Department of Chromosome Biology, Max F. Perutz Laboratories, University of Vienna, Dr. Bohr-Gasse 9, 1030 Vienna, Austria

**Keywords:** *N*-(hydroxybenzylidene)-*N*′-[2,6-dinitro-4-(trifluoromethyl)]phenylhydrazines, radical scavenging activity, DPPH scavenging, GOR scavenging, ABTS scavenging, photosynthetic electron transport

## Abstract

Hydroxybenzylidene hydrazines exhibit a wide spectrum of biological activities. Here, we report synthesis and free radical scavenging activity of nine new *N*-(hydroxybenzylidene)-*N*′-[2,6-dinitro-4-(trifluoromethyl)]phenylhydrazines. The chemical structures of these compounds were confirmed by ^1^H-NMR, ^13^C-NMR, ^19^F-NMR, IR spectroscopy, LC-MS, and elemental analysis. The prepared compounds were tested for their activity to scavenge 2,2-diphenyl-1-picrylhydrazyl (DPPH), galvinoxyl radical (GOR), and 2,2′-azino-bis(3-ethylbenzothiazoline)-6-sulphonic acid (ABTS) radicals. The free radical scavenging activity expressed as SC_50_ values of these compounds varied in a wide range, from a strong to no radical scavenging effect. The most effective radical scavengers were hydroxybenzylidene hydrazines containing three hydroxyl groups in the benzylidene part of their molecules. The prepared compounds were also tested for their activity to inhibit photosynthetic electron transport in spinach chloroplasts. IC_50_ values of these compounds varied in wide range, from an intermediate to no inhibitory effect.

## 1. Introduction

*N*-Hydroxybenzylidene hydrazines, also known as hydrazones, are *N*-arylmethylidene-*N*′-arylhydrazines with a diazamethylidene group C=N-NH [1,2]. The fact that *N*-arylmethylidene-*N*′-arylhydrazines represent an important class of compounds for new drug development motivates researchers to synthesize and test new *N*-arylmethylidene-*N*′-arylhydrazines. These compounds can be prepared from the corresponding aromatic aldehydes and substituted arylhydrazines in alcohol (ethanol, methanol), acetic acid, or another solvents [1,2]. Synthesis without solvents using acidic ionic liquids such as choline chloride and oxalic acid has also been reported [3]. The combination of diazamethylene group with other functional groups leads to compounds with interesting physical and chemical characteristics [4].

The synthesis of novel *N*-arylmethylidene-*N*′-arylhydrazines and their derivatives is of great interest because of their potential use in the biopharmaceutic industry. These compounds possess various biological and pharmacological properties, including antimicrobial, analgesic, antifungal, anti-tubercular, antiviral, anticancer, antimalarial, antihelmintic, anti-trypanosomal, and antischistosomiasis properties. In addition, *N*-arylmethylidene-*N*′-arylhydrazines are used as pigments, dyes, catalysts, ligands in organometallic complexes and polymer stabilizers [5,6,7,8,9,10,11,12]. *N*-arylmethylidene-*N*′-arylhydrazines are also important for the synthesis of heterocyclic compounds such as indoles and pyrazoles [13,14]. Derivatives of benzylidene hydrazine are potent inhibitors of fungal growth with little mammalian cell toxicity, making them promising new targets for future therapeutic development [1]. Several derivatives of *N*-nitrobenzylidene-*N*′-phenylhydrazines exhibit amoebicidal activity with an IC_50_ of 0.84 μM, which represents a sevenfold increase in cell growth inhibition potency with respect to metronidazole (IC_50_ = 6.3 μM) [12]. Several novel 2,4-dinitrophenylhydrazone betulinic acid derivatives showed significant cytotoxicity and selectivity against some tumor cell lines [15]. Due to their strong chemical stability, *N*-arylmethylidene-*N*′-arylhydrazines are also an attractive material in optoelectronics technologies and development of potential chemosensors, optical switching devices, and organic light emission devices (OLEDs) [16]. However, *N*-arylmethylidene-*N*′-arylhydrazines also show adverse effects. Some *N*-arylmethylidene-*N*′-arylhydrazines induce DNA fragmentation [17] and damage photosynthesis in chloroplasts [18,19,20,21].

The main aim of this work was to synthesize new hydroxybenzylidene hydrazines and determine their free radical scavenging activity. We prepared nine new *N*-(hydroxybenzylidene)-*N*′-[2,6-dinitro-4-(trifluoromethyl)]phenylhydrazines with OH groups at different positions of the benzene ring and analyzed their ability to scavenge 2,2-diphenyl-1-picrylhydrazyl (DPPH), galvinoxyl radicals (GOR), and 2,2′-azino-bis(3-ethylbenzothiazoline)-6-sulphonic acid (ABTS).

## 2. Results and Discussion 

### 2.1. Chemistry

**Scheme 1:**
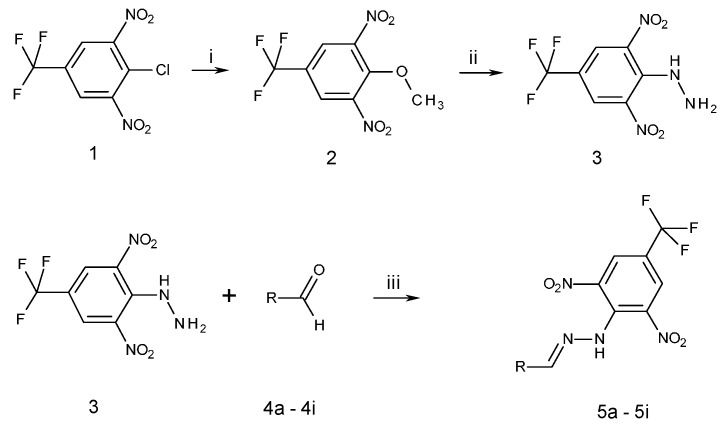
Synthesis of 2,6-dinitro-4-(trifluoromethyl)phenylhydrazine **3** and *N*-(hydroxybenzylidene)-*N*′-[2,6-dinitro-4-(trifluoromethyl)]phenylhydrazines **5a**–**5i**. **5a** R = 4-hydroxyphenyl, **5b** R = 2,3-dihydroxyphenyl, **5c** R = 2,4-dihydroxyphenyl, **5d** R = 2,5-dihydroxyphenyl, **5e** R = 3,5-dihydroxyphenyl, **5f** R = 2,3,4-trihydroxyphenyl, **5g** R = 2,4,6-trihydroxyphenyl, **5h** R = 3,4,5-trihydroxyphenyl, **5i** R = phenyl. Reactants and reaction condition: (i) H_3_C-ONa, methanol, room temperature, 1 h; (ii) H_2_N-NH_2_·H_2_O, EtOH, 0 °C, 1 h; (iii) EtOH, trifluoroacetic acid (TFA), room temperature, 3 h. Structure of all newly prepared compounds was confirmed by ^1^H-NMR, ^13^C-NMR, ^19^F-NMR, IR spectroscopy, and elemental analysis.

All compounds prepared in this study (**5a**–**5i**) contain the 2,4-dinitro-4-(trifluoromethyl)phenyl group and an additional hydroxyphenyl group (**5a**, R = 4-hydroxyphenyl, **5b**, R = 2,3-dihydroxyphenyl, **5c**, R = 2,4-dihydroxyphenyl, **5d**, R = 2,5-dihydroxyphenyl, **5e**, R = 3,5-dihydroxyphenyl, **5f**, R = 2,3,4-trihydroxyphenyl, **5g**, R = 2,4,6-trihydroxyphenyl, **5h**, R = 3,4,5-trihydroxyphenyl, and **5i**, R = phenyl) (Scheme 1).

The starting compounds for the synthesis of hydroxybenzylidene hydrazines **5a**–**5i** were 2,6-dinitro-4-(trifluoromethyl)phenylhydrazine **3** and aromatic aldehydes **4a**–**4i** (Scheme 1). The starting compound for the preparation of 2-methoxy-1,3-dinitro-5-(trifluoromethyl)benzene **2** (Scheme 1) was 2-chloro-1,3-dinitro-4-(trifluoromethyl)benzene **1**. The yield of the compound **2** was 76%, M.p. 61 °C [22].

For the synthesis of compounds **5a**–**5i**, we used trifluoroacetic acid (TFA) as acidic catalyst and ethanol as solvent. The reaction time was 3–4 h, reaction temperatures were 20–25 °C, and yields were 70–85%. The purity of prepared compounds and the course of reactions were monitored by TLC. The crude hydrazines **5a–5i** were purified by column chromatography on silica gel in hexane/ethylacetate (4:1) as the mobile phase. All prepared hydroxybenzylidene hydrazines **5a**–**5i** as well as their solutions in organic solvents were dark red. The dark red color changed to dark blue upon the increase in pH, suggesting that these compounds are sensitive to pH. The melting points of these compounds are relatively high (226–280 °C). The chemical structures of prepared compounds were confirmed by ^1^H-NMR, ^13^C-NMR, ^19^F-NMR, IR spectroscopy, LC-MS and elemental analysis. Elemental analyses agreed with theoretical values (±0.3). The IR spectra revealed several characteristic absorption bands. Two absorption bands were observed in the region stretching NO_2_ vibration. The more intense band appearing at higher wave numbers (1538–1503 cm^−1^) corresponds to assymetric NO_2_ vibrations, and the less intense one, appearing at a lower wave number (1279–1257 cm^−1^), corresponds to symmetric vibrations. The absorption band at 3277–3257 cm^−1^ corresponds to stretching N–H vibrations. The wave number of this absorption band is affected by the mesomeric effect of OH groups on the benzene ring. The absorption band at 1619–1616 cm^−1^ corresponds to stretching vibrations in the C=N group. The presence of CF_3_ group in molecules was confirmed by ^19^F-^13^C splitting to quartet in ^13^C NMR spectra of corresponding derivatives [4′ (115.82–116.71 ppm, ^2^*J* = 35.1–35.4 Hz); 3′,5′ (127.189–127.72 ppm, ^3^*J* = 3.2–3.5 Hz); CF3 (122.67–123.20 ppm, ^1^*J* = 271.3–271.6 Hz)]. 

The double bond between C and N in C=N–NH– group of benzylidene hydrazines allows for the formation of two stereoisomers, namely (E) and (Z). The stereoisomerism of these compounds has not been studied in detail, but we predict that (E) is the more abundant form [2,23].

### 2.2. Free Radical Scavenging Activity Assay

SC_50_ values for scavenging DPPH, GOR, and ABTS radicals are shown in the Table 1. These results suggest that the majority of studied hydroxybenzylidene hydrazines exhibit free radical scavenging activity. Almost all studied benzylidene hydrazines (with the exception of compound **5i**) scavenged ABTS radical in a water solution. The most effective scavengers of ABTS radicals were compounds **5b** and **5e** with two OH groups in positions 2, 3 and 3, 5, respectively. Hydroxybenzylidene hydrazines with three hydroxyl groups (**5b**, **5f**, **5h** and **5g**) also scavenged ABTS radicals. The most effective scavengers of DPPH radicals were hydroxybenzylidene hydrazines with three OH groups (**5h** and **5f**). The molecules with two OH groups (**5b**, **5c**, **5g**) were weaker scavengers of DPPH radicals. Hydroxybenzylidene hydrazine with one OH group (**5a**) exhibited very weak scavenger activity, and the compound with OH groups in positions 3 and 5 (**5e**) as well as the compound without OH group (**5i**) did not scavenge DPPH radicals.

The compound with three OH groups (**5f**) in positions 2, 3, and 4 was the most effective scavenger of GOR radicals. The compounds **5b**, **5d**, **5g**, and **5h** exhibited moderate scavenger activity, and the compound **5a** (with one OH group) exhibited very low activity to scavenge GOR radicals. Similarly as observed with DPPH radicals, the compounds **5e** and **5i** showed no scavenging of GOR.

Taken together, we conclude that molecules with three hydroxyl groups (**5b**, **5f**, and **5h**) exhibited high free radical scavenging activities. The most effective compound was the compound **5f**, which contains OH groups in positions 2, 3, and 4. Interestingly, the compound with no hydroxyl group (**5i**) showed no scavenging activity. Some of the studied compounds (**5f** and **5h**) exhibited higher free radical scavenging activity than that reported for ascorbic acid (SC_50_ = 12.55 μmol/dm^3^) [24], resveratrol (SC_50_ = 26.37 μmol/dm^3^) [25], and esculetin (SC_50_ = 8.64 μmol/dm^3^) [26]. We speculate that the ability to release hydrogen atom or proton from hydroxyl groups of the hydroxybenzylidene hydrazine molecule contributes to the mechanism of radical scavenging of the studied hydroxybenzylidene hydrazines. 

**Figure 1:**
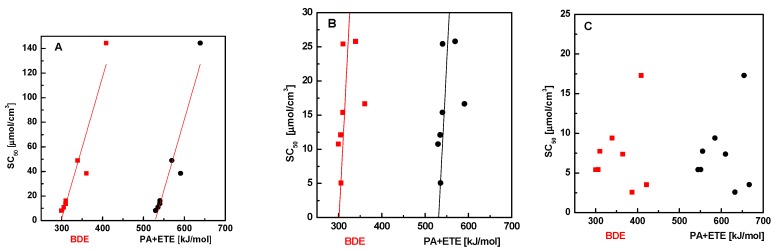
Dependence of SC_50_ of DPPH (**A**), GOR (**B**), and ABTS (**C**) scavenging on BDE (red squares) or PA + ETE (black circles).

Next, we tried to find a possible correlation between free radical scavenging activity and energy necessary for releasing of hydrogen atom or proton. We used the method PM6 (see the Experimental Section) to calculate the energy associated with the release of hydrogen atom (bond dissociation enthalpy, BDE), the proton relaxation (proton dissociation enthalpy, PDE), release of electron (ionization potential, IP), and the energy associated with the combined transfer of the electron and proton (ETE and PA, respectively) for the studied benzylidene hydrazines. The values of these energies in methanol or in water are presented in Tables S1–S8. Figure 1 shows the dependence of SC_50_ of DPPH, GOR, and ABTS scavenging on the sum of PA + ETE values and BDE, respectively. The results presented in the Figure 1A,B suggest that the ability of scavenging of DPPH and GOR radicals at higher BDE and PE + ETE enthalpy decreases—the dependence is almost linear (the square deviation r^2^ = 0.87 for DPPH and r^2^ = 0.70 for GOR). This tendency is confirmed by the fact that the inactive substances (**5a** and **5e**) have the greatest values of BDE and PA + ETE (Table 1). On the other hand, the dependence of the scavenging of ABTS radicals on BDE or PA + ETE shows no dependence (Figure 1C).

### 2.3. Inhibition of Photosynthetic Electron Transport (PET) in Spinach Chloroplasts

The prepared compounds were also tested for their activity to inhibit PET in spinach chloroplasts. IC_50_ values of these compounds varied in a wide range, from an intermediate (14.7 μmol/dm^3^) to no inhibitory effect (Table 1). The most effective were compounds **5c** (IC_50_ = 14.7 μmol/dm^3^) and **5b** (IC_50_ = 42.8 μmol/dm^3^). These activities are relatively low as compared to the values reported for the classical herbicide diuron (3-(3,4-dichlorophenyl)-1,1-dimethylurea; DCMU) with an IC_50_ of 1.9 μmol/dm^3^ [27] and are unlikely to be of industrial interest.

## 3. Materials and Methods

### 3.1. General Information 

2-Chloro-1,3-dinitro-4-(trifluoromethyl)benzene **1** (Scheme 1), galvinoxyl free radical (GOR), and organic solvents were purchased from Alfa Aesar (Ward Hill, MA, USA) and used without further purification. 2,2-Diphenyl-1-picrylhydrazyl (DPPH) and 2,2′-azino-bis(3-ethylbenzothiazoline-6-sulfonic acid) diammonium salt (ABTS) was purchased from Sigma-Aldrich (St. Louis, MO, USA). Methyl alcohol p.a., TRIS, MgCl_2_, saccharose and dimethylsulfoxide p.a. (DMSO) were purchased from Centralchem (Bratislava, Slovakia).

Melting points were determined on a Boetius apparatus and are uncorrected. IR spectra were obtained on a NICOLET iS50 FT-IR spectrophotometer using an ATR technique in the region 4000–400 cm**^−^**^1^. Elemental analyses were obtained on an Elemental Analyzer Carlo Erba CHNS-OEA 1108. NMR spectra were performed on a Spectrometer Varian VNMRS 300 MHz (300 MHz for ^1^H, 75 MHz for ^13^C, 282 MHz for ^19^F) and on a Spectrometer Varian VNMRS 600 MHz (600 MHz for ^1^H and 150 MHz for ^13^C) in DMSO-*d*_6_, with tetramethylsilane (TMS) as an internal standard. The purity of prepared compounds and the course of reactions were checked on Merck TLC Silica gel 60 F_254_ plates in ethyl acetate–*n*-hexane as the mobile phase. The numbering of atoms for the evaluation of NMR spectra of measured compounds **3** and **5a**–**5i** is given in the formulae. Absorption spectra were recorded by a Genesis 6 spectrophotometer (Thermo–Scientific, Waltham, MA, USA). FTIR spectra (in solid phase) were recorded on a Nicolet 6700 spectrometer (Thermo–Scientific (Nicolet), Waltham, MA, USA) using the ATR technique.

MS spectra were recorded by LC-MS spectrometer consisting of an Agilent 1200 HPLC (Walbron, Germany), with an MSD 6110 MS detector (Agilent Technologies, Santa Clara, CA, USA).

### 3.2. Synthesis 

#### 3.2.1. Synthesis of 2,6-Dinitro-4-(Trifluoromethyl)Phenylhydrazine (**3**)

A solution of hydrazine monohydrate (0.75 g, 15 mmol) in anhydrous ethanol (5 mL) was added dropwise to a solution of 2-methoxy-1,3-dinitro-5-(trifluoromethyl)benzene 2 (3.2 g, 12 mmol) in anhydrous ethanol (17 mL) under an argon atmosphere. The reaction mixture was stirred at 0 °C for 1 h and monitored by TLC. The solvent was removed under vacuum, and crude product was purified by column chromatography on silica gel in hexane/ethylacetate (4:1) as the mobile phase. This resulted in 2.8 g (88%) of 2,6-dinitro-4-(trifluoromethyl)phenylhydrazine 3 (yellow solid), M.p. 125–126 °C. The previously published M.p. for this compound is 124 °C [23].


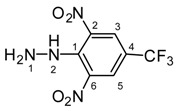


*2,6-dinitro-4-(trifluoromethyl)phenylhydrazine* (**3**). Yield 88%; red solid, M.p. 125–126 °C, Anal. Calcd. for C_7_H_5_F_3_N_4_O_4_ (266.14) C, 31.59; H, 1.89: N, 21.05. Found: C, 31.71; H, 1.74; N, 20.86%. IR: 3248, ʋ(N–H); 1636, ʋ(C=N); 1536, ʋ_as_(NO_2_); 1263, ʋ_s_(NO_2_); 1118, ʋ(CF_3_). ^1^H NMR (300 MHz, DMSO): δ = 9.65 (s, 1H, NH-2), 8.40 (s, 2H, H-3, H-5), 4.84 (s, 2H, NH-1). ^13^C NMR (75 MHz, DMSO): δ = 142.97, 136.75, 127.87 (q, *J* = 3.5 Hz), 123.32 (q, *J* = 271.0 Hz), 113.76 (q, *J* = 35.3 Hz); negative LC-MS *m*/*z*: 265.0 [M − H]^−^ calc. for C_7_H_4_F_3_N_4_O_4_^−^, 265.018, found 265.0.

#### 3.2.2. Synthesis of *N*-Hydroxybenzylidene-*N*′-[2,6-Dinitro-4-(Trifluoromethyl)]Phenylhydrazines (**5a**–**5h**) and *N*-(Benzylidene)-*N*′-[2,6-Dinitro-4-(Trifluoromethyl)]Phenylhydrazine (**5i**)

A solution of 2,6-dinitro-4(trifluoromethyl)phenylhydrazine 3 (1 mmol) in ethanol (3 mL) was added to a solution of aldehydes **4a**–**4i** in ethanol (5 mL) and trifluoroacetic acid (0.5 mL). The reaction mixture was stirred for 3–4 h at room temperature in argon atmosphere and monitored by TLC. The reaction mixture was cooled to 0 °C, and the red solid product was filtered off, washed with ethanol/ether (1:5), and dried. The product was purified by column chromatography on silica gel in hexane/ethylacetate (3:1) as the mobile phase.


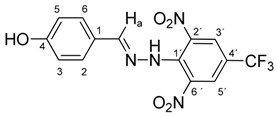


*N-(4-Hydroxybenzylidene)-N′-[2,6-dinitro-4-(trifluoromethyl)]phenylhydrazine* (**5a**). Yield 72%; red solid, M.p. 246–248 °C, Anal. Calcd. for C_7_H_9_F_3_N_4_O_5_ (370.24) C, 45.42; H, 2.45: N, 15.13. Found: C, 45.30; H, 2.38; N, 15.10%. IR: 3507, ʋ(O–H); 3277, ʋ(N–H); 1636, ʋ(C=N); 1537, ʋ_as_(NO_2_); 1261, ʋ_s_(NO_2_); 1125, ʋ(CF_3_). ^1^H NMR (600 MHz, DMSO): δ = 11.46 (s, 1H, NH), 10.05 (s, 1H, OH), 8.53 (s, 2H, H-3′, H-5′), 8.41 (s, 1H, H-a), 7.39 (d, *J* = 8.6 Hz, 2H, H-2, H-6), 6.84 (d, *J* = 8.6 Hz, 2H, H-3, H-5). ^13^C NMR (151 MHz, DMSO): δ = 160.34, 149.53, 137.47, 136.16, 129.54, 127.64 (q, *J* = 3.3 Hz), 125.11, 123.17 (q, *J* = 271.5 Hz), 116.37 (q, *J* = 35.1 Hz), 116.32; negative LC-MS *m*/*z*: 369.0 [M − H]^−^ calc. for C_7_H_8_F_3_N_4_O_5_^−^, 369.045, found 369.0.


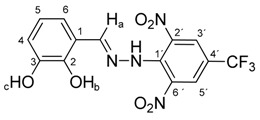


*N-(2,3-Dihydroxybenzylidene)-N′-[2,6-dinitro-4-(trifluoromethyl)]phenylhydrazine* (**5b**). Yield 79%; red solid, M.p. 226–228 °C, Anal. Calcd. for C_14_H_9_F_3_N_4_O_6_ (386.24) C, 43.54; H, 2.35: N, 14.51. Found: C, 43.64; H, 2.39; N, 14.63%. IR: 3528, 3377 ʋ(O–H); 3274, ʋ(N–H); 1633, ʋ(C=N); 1537, ʋ_as_(NO_2_); 1279, ʋ_s_(NO_2_); 1125, ʋ(CF_3_). ^1^H NMR (600 MHz, DMSO): δ = 11.56 (s, 1H, NH), 9.63 (s, 1H, OH-b), 9.03 (s, 1H, OH-c), 8.79 (s, 1H, H-a), 8.51 (s, 2H, H-3′, H-5′), 6.93 (dd, *J* = 7.9, 1.5 Hz, 1H, H-6), 6.81 (dd, *J* = 7.9, 1.5 Hz, 1H, H-4), 6.65 (dd, *J* = 7.9 Hz, 1H, H-5). ^13^C NMR (151 MHz, DMSO): δ = 146.29, 146.19, 146.15, 137.71, 136.11, 127.63 (q, *J* = 3.4 Hz), 123.15 (q, *J* = 271.4 Hz), 121.24, 119.81, 117.36, 116.71 (q, *J* = 35.4 Hz), 116.44; negative LC-MS *m*/*z*: 385.0 [M − H]^−^ calc. for C_14_H_8_F_3_N_4_O_6_^−^, 385.04, found 385.0.


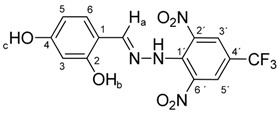


*N-(2,4-Dihydroxybenzylidene)-N′-[2,6-dinitro-4-(trifluoromethyl)]phenylhydrazine* (**5c**). Yield 73%; red solid, M.p. 229–230 °C, Anal. Calcd. for C_14_H_9_F_3_N_4_O_6_ (386.24) C, 43.54; H, 2.35: N, 14.51. Found: C, 43.66; H, 2.24; N, 14.40%. IR: 3528, 3377 ʋ(O–H); 3274, ʋ(N–H); 1633, ʋ(C=N); 1537, ʋ_as_(NO_2_); 1279, ʋ_s_(NO_2_); 1125, ʋ(CF_3_). ^1^H NMR (600 MHz, DMSO): δ = 11.46 (s, 1H, NH), 9.94 (s, 1H, OH-b), 9.88 (s, 1H, OH-c), 8.63 (s, 1H, H-a), 8.48 (s, 2H, H-3′, H-5′), 7.27 (d, *J* = 8.6 Hz, 1H, H-6), 6.31 (d, *J* = 2.2 Hz, 1H, H-3), 6.28 (dd, *J* = 8.6, 2.2 Hz, 1H, H-5). ^13^C NMR (151 MHz, DMSO): δ = 161.69, 158.89, 146.59, 137.36, 136.07, 127.82, 127.67 (q, *J* = 3.2 Hz), 123.20 (q, *J* = 271.4 Hz), 115.93 (q, *J* = 35.3 Hz), 112.09, 108.66, 102.73; negative LC-MS *m*/*z*: 385.0 [M − H]^−^ calc. for C_14_H_8_F_3_N_4_O_6_^−^, 385.04, found 385.0.


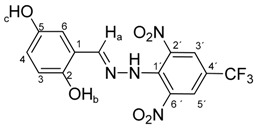


*N-(2,5-Dihydroxybenzylidene)-N′-[2,6-dinitro-4-(trifluoromethyl)]phenylhydrazine* (**5d**). Yield 83%; red solid, M.p. 233–234 °C, Anal. Calcd. for C_14_H_9_F_3_N_4_O_6_ (386.24) C, 43.54; H, 2.35: N, 14.51. Found: C, 43.41; H, 2.42; N, 14.62%. IR: 3517, 3346 ʋ(O–H); 3257, ʋ(N–H); 1635, ʋ(C=N); 1536, ʋ_as_(NO_2_); 1270, ʋ_s_(NO_2_); 1129, ʋ(CF_3_). ^1^H NMR (600 MHz, DMSO): δ = 11.55 (s, 1H, NH), 9.40 (s, 1H, OH-b), 8.95 (s, 1H, OH-c), 8.73 (s, 1H, H-a), 8.54 (s, 2H, H-3′, H-5′), 6.85 (d, *J* = 2.1 Hz, 1H, H-6), 6.73 (s, 2H, H-4, H-3). ^13^C NMR (151 MHz, DMSO): δ = 150.42, 150.34, 146.27, 137.74, 136.10, 127.64 (q, *J* = 3.5 Hz), 123.15 (q, *J* = 271.6 Hz), 120.70, 120.21, 117.42, 116.71 (q, *J* = 35.3 Hz), 111.37; negative LC-MS *m*/*z*: 385.1 [M − H]^−^ calc. for C_14_H_8_F_3_N_4_O_6_^−^, 385.04, found 385.1.


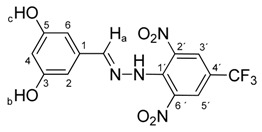


*N-(3,5-Dihydroxybenzylidene)-N′-[2,6-dinitro-4-(trifluoromethyl)]phenylhydrazine* (**5e**). Yield 78%; red solid, M.p. 264–266 °C, Anal. Calcd. for C_14_H_9_F_3_N_4_O_6_ (386.24) C, 43.54; H, 2.35: N, 14.51. Found: C, 43.66; H, 2.23; N, 14.38%. IR: 3539, 3347 ʋ(O–H); 3270, ʋ(N–H); 1637, ʋ(C=N); 1536, ʋ_as_(NO_2_); 1268, ʋ_s_(NO_2_); 1126, ʋ(CF_3_). ^1^H NMR (300 MHz, DMSO): δ = 11.45 (s, 1H, NH), 9.49 (s, 2H, OH-b, OH-c), 8.55 (s, 2H, H-3′, H-5′), 8.34 (s, 1H, H-a), 6.42 (d, *J* = 2.1 Hz, 2H, H-2, H-6), 6.32 (dd, *J* = 2.1 Hz, 1H, H-4). ^13^C NMR (75 MHz, DMSO): δ = 158.63, 149.63, 137.33, 135.66, 135.20, 127.19 (q, *J* = 17.8 Hz), 122.67 (q, *J* = 271.5 Hz), 116.60 (q, *J* = 35.4 Hz), 105.48, 105.01; negative LC-MS *m*/*z*: 385.0 [M − H]^−^ calc. for C_14_H_8_F_3_N_4_O_6_^−^, 385.04, found 385.0.


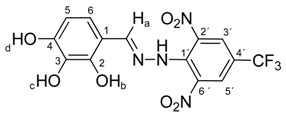


*N-(2,3,4-Dihydroxybenzylidene)-N′-[2,6-dinitro-4-(trifluoromethyl)]phenylhydrazine* (**5f**). Yield 85%; red solid, M.p. 232–233 °C, Anal. Calcd. for C_14_H_9_F_3_N_4_O_7_ (402.24) C, 41.80; H, 2.26: N, 13.93. Found: C, 41.70; H, 2.18; N, 14.05%. IR: 3532, 3481, 3359 ʋ(O–H); 3272, ʋ(N–H); 1633, ʋ(C=N); 1530, ʋ_as_(NO_2_); 1269, ʋ_s_(NO_2_); 1128, ʋ(CF_3_). ^1^H NMR (300 MHz, DMSO): δ = 11.48 (s, 1H, NH), 9.70 (s, 1H, OH-b), 9.03 (s, 1H, OH-d), 8.67 (s, 1H, OH-c), 8.54 (s, 1H, H-a), 8.50 (s, 2H, H-3′, H-5′), 6.85 (d, *J* = 8.6 Hz, 1H, H-6), 6.37 (d, *J* = 8.6 Hz, 1H, H-5). ^13^C NMR (151 MHz, DMSO): δ = 160.34, 149.53, 137.47, 136.16, 129.54, 127.64 (q, *J* = 3.3 Hz), 125.11, 123.17 (q, *J* = 271.5 Hz), 116.37 (q, *J* = 35.1 Hz), 116.32; negative LC-MS *m*/*z*: 401.0 [M − H]^−^ calc. for C_14_H_8_F_3_N_4_O_7_^−^, 401.035, found 401.0.


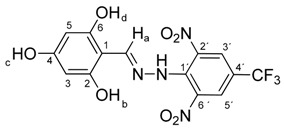


*N-(2,4,6-Dihydroxybenzylidene)-N′-[2,6-dinitro-4-(trifluoromethyl)]phenylhydrazine* (**5g**). Yield 84%; red solid, M.p. 238–240 °C, Anal. Calcd. for C_14_H_9_F_3_N_4_O_7_ (402.24) C, 41.80; H, 2.26: N, 13.93. Found: C, 41.96; H, 2.30; N, 13.80%. IR: 3323 ʋ(O–H); 3262, ʋ(N–H); 1633, ʋ(C=N); 1532, ʋ_as_(NO_2_); 1263, ʋ_s_(NO_2_); 1126, ʋ(CF_3_). ^1^H NMR (300 MHz, DMSO): δ = 11.44 (s, 1H, NH), 10.02 (s, 1H, OH-c), 9.83 (s, 2H, OH-b, OH-d), 8.89 (s, 1H, H-a), 8.54 (s, 2H, H-3′, H-5′), 5.86 (s, 2H, H-3, H-5). ^13^C NMR (75 MHz, DMSO): δ = 162.57, 159.57, 150.75 (q, *J* = 14.5 Hz), 137.13, 135.08, 127.72 (q, *J* = 18.9 Hz), 122.68 (q, *J* = 271.5 Hz), 115.91 (q, *J* = 35.4 Hz), 98.57, 94.47; negative LC-MS *m*/*z*: 401.0 [M − H]^−^ calc. for C_14_H_8_F_3_N_4_O_7_^−^, 401.035, found 401.0.


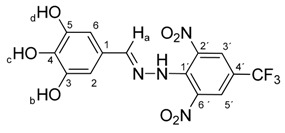


*N-(3,4,5-Trihydroxybenzylidene)-N′-[2,6-dinitro-4-(trifluoromethyl)]phenylhydrazine* (**5h**). Yield 71%; red solid, M.p. 279–280 °C, Anal. Calcd. for C_14_H_9_F_3_N_4_O_7_ (402.24) C, 41.80; H, 2.26: N, 13.93. Found: C, 41.62; H, 2.15; N, 13.80%. IR: 3428 ʋ(O–H); 3266, ʋ(N–H); 1634, ʋ(C=N); 1537, ʋ_as_(NO_2_); 1265, ʋ_s_(NO_2_); 1125, ʋ(CF_3_). ^1^H NMR (300 MHz, DMSO): δ = 11.37 (s, 1H, NH), 9.15 (s, 2H, OH-b, OH-d), 8.78 (s, 1H, OH-c), 8.53 (s, 2H, H-3′, H-5′), 8.26 (s, 1H, H-a), 6.53 (s, 2H, H-2, H-6). ^13^C NMR (75 MHz, DMSO): δ = 150.27, 146.09, 137.01, 136.57, 135.66, 127.23 (q, *J* = 3.5 Hz), 123.76, 122.73 (q, *J* = 271.5 Hz), 115.82 (q, *J* = 35.4 Hz), 106.85; negative LC-MS *m*/*z*: 401.0 [M − H]^−^ calc. for C_14_H_8_F_3_N_4_O_7_^−^, 401.035, found 401.0.


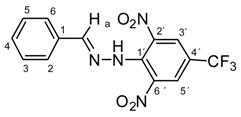


*N-(Benzylidene)-N′-[2,6-dinitro-4-(trifluoromethyl)]phenylhydrazine* (**5i**). Yield 70%; red solid, M.p. 231–232 °C, Anal. Calcd. for C_14_H_9_F_3_N_4_O_4_ (354.24) C, 47.47; H, 2.56: N, 15.82. Found: C, 47.65; H, 2.48; N, 15.71%. IR: 3265, ʋ(N–H); 1631, ʋ(C=N); 1538, ʋ_as_(NO_2_); 1267, ʋ_s_(NO_2_); 1121, ʋ(CF_3_). ^1^H NMR (600 MHz, DMSO): δ = 11.57 (s, 1H, NH), 8.57 (s, 2H, H-3′, H-5′), 8.53 (s, 1H, H-a), 7.55 (dd, *J* = 7.7, 1.4 Hz, 2H, H-2, H-6), 7.50 – 7.42 (m, 3H, H-3, H-4, H-5). ^13^C NMR (151 MHz, DMSO): δ = 149.02, 137.84, 136.05, 134.15, 130.94, 129.44, 127.64 (q, *J* = 3.4 Hz), 127.58, 123.10 (q, *J* = 271.6 Hz), 117.29 (q, *J* = 35.3 Hz); negative LC-MS *m*/*z*: 353.0 [M − H]^−^ calc. for C_14_H_9_F_3_N_4_O_4_^−^, 353.05, found 353.0.

### 3.3. Free Radical Scavenging Activity Assay

The free radical scavenging activity of the prepared hydroxybenzylidene hydrazines was carried out according to our previous work [25]. Various amounts of tested compounds were added into a methanol solution of DPPH or GOR and the final DPPH or GOR concentration was kept constant (*c* = 10^−4^ mol·dm^−3^) or into a water solution of ABTS. The free radical scavenging activity was evaluated using the values SC_50_, i.e., the concentration of the studied compound, which causes a 50% decrease in absorbance at 517 nm (for DPPH), 862 nm (for GOR), or 734 nm (for ABTS) as compared to the control sample. Methanol or water was used as a blank. All samples were measured in triplicate. Standard square deviations were in a range from 0.91 to 0.99.

### 3.4. Photosynthetic Electron Transport (PET) Study

PET was monitored in spinach chloroplasts prepared according to our previous work [4,27]. PET through PSII was monitored by the Hill reaction with DCPIP as an artificial electron acceptor. DCPIP photoreduction was determined spectrophotometrically. The chlorophyll (Chl) concentration in these experiments was 30 mg/dm^3^. The inhibitory activities of the studied compounds were expressed by IC_50_ values, i.e., molar concentrations of the compounds causing a 50% decrease of absorbance at 600 nm compared to the control sample. Each sample was measured in triplicate and standard square deviations were in the 0.88–0.94 range.

### 3.5. Molecular Calculations

The prepared hydroxybenzylidene hydrazines, their anions and radicals were studied using the quantum chemical method PM6 [28], which is part of the program MOPAC2012 [29]. Optimal structures of compounds were calculated (keyword PRECISE). The effect of solvents on the above mentioned compounds were studied by COSMO-method [30], which is also part of MOPAC2012 [31]. Ionization potentials and enthalpy of formations used for the calculation of PDE, BDE, PA, and ETE according to our previous work [25].

## Figures and Tables

**Table 1:** Antiradical and PET inhibition activities of studied hydroxybenzilidene hydrazines expressed as SC_50_ and IC_50_ values, respectively.

Compound	SC_50_ μM DPPH	SC_50_ μM GOR	SC_50_ μM ABTS	IC_50_ μM PET Inhibition	BDE	PA + ETE	BDE	PA + ETE
					(kJ/mol) in CH_3_OH	(kJ/mol) in CH_3_OH	(kJ/mol) in Water	(kJ/mol) in Water
**5a**	144.4	168.1	17.3	1003	408.8	638.9	408.7	654.1
**5b**	16.2	25.4	2.6	42.8	387.2	540.2	387.0	632.2
**5c**	48.9	25.8	9.4	14.7	339.1	569.2	339.3	584.7
**5d**	13.7	15.4	7.8	93	309.8	539.9	309.8	555.2
**5e**	NDA ^1^	NDA ^1^	3.5	NDA ^1^	421.3	651.7	421.1	666.5
**5f**	10.8	5.1	5.4	NDA ^1^	305.3	535.5	305.5	550.8
**5g**	38.5	16.7	7.4	263	351.6	590.8	364.4	612.3
**5h**	8.2	10.8	5.4	NDA ^1^	299.5	529.7	299.9	545.2
**5i**	NDA ^1^	NDA ^1^	182.9	344				

^1^ NDA (no detectable activity).

## Supplementary Materials

The following are available online: Table S1: Proton dissociation energy of prepared *N*-hydroxybenzylidene hydrazines in methanol; Table S2: Dissociation energy of hydrogen and electron of prepared *N*-hydroxybenzylidene hydrazines in methanol; Table S3: Proton affinity of prepared *N*-hydroxybenzylidene hydrazines in methanol; Table S4: Electron transfer enthalpy of prepared *N*-hydroxybenzylidene hydrazines in methanol; Table S5: Proton dissociation energy of prepared *N*-hydroxybenzylidene hydrazines in water; Table S6: Dissociation energy of hydrogen and electron of prepared *N*-hydroxybenzylidene hydrazines in water; Table S7: Proton affinity of prepared *N*-hydroxybenzylidene hydrazines in water; Table S8: Electron transfer enthalpy of prepared *N*-hydroxybenzylidene hydrazines in water.
