# Supplementary material for: Synthesis and Free Radical Scavenging Activity of New Hydroxybenzylidene Hydrazines
Source: Molecules. 2017 May 29;22(6):894. doi: 10.3390/molecules22060894 (PMC6152720; doi:10.3390/molecules22060894)
Supplement: Supplementary file 1 [file molecules-22-00894-s001.pdf]

Supplementary Materials: Synthesis and Free Radical Scavenging Activity of New Hydroxybenzylidene Hydrazines

Frantisek Sersen, Fridrich Gregan, Peter Kotora, Jarmila Kmetova, Juraj Filo, Dušan Loos and Juraj Gregan

Table S1. Proton dissociation energy of prepared *N*-hydroxybenzylidene hydrazines in methanol. The position of hydroxyl group in bezilidene ring is indicated (pos.i).

Compound	PDE (kJ/mol)				
	pos.2	pos.3	pos.4	pos.5	pos.6
**5a**			202.5		
**5b**	251.8	183.0			
**5c**	141.2		145.0		
**5d**	127.0			136.4	
**5e**		110.2		110.8	
**5f**	100.5	92.3	100.4		
**5g**	154.7		162.5		161.4
**5h**		125.4	109.4	125.2	

Table S2. Dissociation energy of hydrogen and electron of prepared *N*-hydroxybenzylidene hydrazines in methanol.

Compound	BDE (kJ/mol)					IP
	pos.2	pos.3	pos.4	pos.5	pos.6	
**5a**			408.8			881.1
**5b**	456.0	387.2				888.0
**5c**	339.1		342.8			880.2
**5d**	309.8			319.3		865.3
**5e**		421.3		421.9		918.8
**5f**	313.4	305.3	313.3			887.8
**5g**	351.6		359.5		358.3	884.0
**5h**		315.6	299.5	315.4		884.7

Table S3. Proton affinity of prepared *N*-hydroxybenzylidene hydrazines in methanol.

Compound	PA (kJ/mol)				
	pos.2	pos.3	pos.4	pos.5	pos.6
**5a**			249.7		

**5b**	325.8	251.4			
**5c**	229.3		236.0		
**5d**	241.7			268.0	
**5e**		249.4		248.9	
**5f**	219.0	251.5	225.5		
**5g**	225.5		226.3		220.0
**5h**		237.1	245.3	239.6	

Table S4. Electron transfer enthalpy of prepared *N*-hydroxybenzylidene hydrazines in methanol.

**Compound**	**ETE (kJ/mol)**				
	pos.2	pos.3	pos.4	pos.5	pos.6
**5a**			389.2		
**5b**	360.26	365.8			
**5c**	339.9		337.0		
**5d**	298.2			281.3	
**5e**		402.0		403.2	
**5f**	324.6	284.0	318.0		
**5g**	356.2		363.3		368.4
**5h**		308.6	284.4	305.9	

Table S5. Proton dissociation energy of prepared *N*-hydroxybenzylidene hydrazines in water.

	**PDE (kJ/mol)**				
**Compound**	pos.2	pos.3	pos.4	pos.5	pos.6
**5a**			161.7		
**5b**	212.0	142.7			
**5c**	100.5		104.0		
**5d**	86.9			103.1	
**5e**		173.7		174.13	
**5f**	61. 2	53.1	61.1		
**5g**	117.4		121.1		120.0
**5h**		85.1	69.3	85.0	

Table S6. Dissociation energy of hydrogen and electron of prepared *N*-hydroxybenzylidene hydrazines in water.

**Compound**		**BDE (kJ/mol)**					
		pos.2	pos.3	pos.4	pos.5	pos.6	**IP**
**5a**				408.7			880.6

**5b**		456.2	387.0				887.7
**5c**		339.3		342.8			879.9
**5d**		309.8			318.9		865.7
**5e**			421.1		421.5		916.1
**5f**		313.6	305.5	313.4			887.4
**5g**		364.4		368.0		366.9	899.5
**5h**			315.8	299.9	315.6		884.8

Table S7. Proton affinity of prepared *N*-hydroxybenzylidene hydrazines in water.

**Compound**	**PA (kJ/mol)**				
	pos.2	pos.3	pos.4	pos.5	pos.6
**5a**			199.7		
**5b**	276.6	201. 1			
**5c**	179.8		186.2		
**5d**	192.4			216.9	
**5e**		197.9		198.1	
**5f**	169.6	201.3	175.9		
**5g**	183.8		185.1		177.6
**5h**		186.7	195.8	189.1	

Table S8. Electron transfer enthalpy of prepared *N*-hydroxybenzylidene hydrazines in water.

**Compound**	**ETE (kJ/mol)**				
	pos.2	pos.3	pos.4	pos.5	pos.6
**5a**			454.4		
**5b**	425.0	431.2			
**5c**	404.9		401.9		
**5d**	362.8			347.4	
**5e**		468.6		468.8	
**5f**	389.3	349.5	382.8		
**5g**	425.9		428.3		434.7
**5h**		374.4	349.4	371.8	
